# Effectiveness of cinacalcet treatment for secondary hyperparathyroidism on hospitalization: Results from the MBD-5D study

**DOI:** 10.1371/journal.pone.0216399

**Published:** 2019-05-29

**Authors:** Shinji Asada, Kazuki Yoshida, Shingo Fukuma, Takanobu Nomura, Michihito Wada, Yoshihiro Onishi, Noriaki Kurita, Masafumi Fukagawa, Shunichi Fukuhara, Tadao Akizawa

**Affiliations:** 1 Medical Affairs Department, Kyowa Hakko Kirin, Chiyoda-ku, Tokyo, Japan; 2 Departments of Epidemiology and Biostatistics, Harvard T.H. Chan School of Public Health, Boston, Massachusetts, United States of America; 3 Human Health Sciences, Kyoto University Graduate School of Medicine, Kyoto, Japan; 4 The Keihanshin Consortium for Fostering the Next Generation of Global Leaders in Research (K-CONNEX), Kyoto, Japan; 5 Institute for Health Outcomes and Process Evaluation Research (iHope International), Kyoto, Japan; 6 Center for Innovative Research for Communities and Clinical Excellence (CiRC(2)LE), Fukushima Medical University, Fukushima, Japan; 7 Department of Innovative Research and Education for Clinicians and Trainees (DiRECT), Fukushima Medical University Hospital, Fukushima, Japan; 8 Division of Nephrology, Endocrinology and Metabolism, Tokai University School of Medicine, Tokyo, Japan; 9 Department of Healthcare Epidemiology, School of Public Health, Kyoto University Faculty of Medicine, Kyoto, Japan; 10 Showa University, Shinagawa-ku, Tokyo, Japan; Tokushima University Graduate School, JAPAN

## Abstract

**Objectives:**

To elucidate the effect of cinacalcet use on all-cause and cause-specific hospitalization outcomes using a prospective cohort of maintenance hemodialysis patients.

**Methods:**

We used data from a prospective cohort of Japanese hemodialysis patients with secondary hyperparathyroidism and examined baseline characteristics as well as longitudinal changes. All patients were cinacalcet-naïve at study enrollment. Further, we used a marginal structural model to account for time-varying confounders on cinacalcet initiation and hospitalization outcomes, and an Andersen-Gill–type recurrent event model to account for any recurring events of hospitalization in the outcome analysis using the weighted dataset.

**Results:**

Among the 3,276 patients, cinacalcet treatment was initiated in 1,384 patients during the entire follow-up. Cinacalcet users were slightly younger, included more patients with chronic glomerulonephritis and fewer patients with diabetes, were more likely to have a history of parathyroidectomy, and were more often used receiving vitamin D receptor activator, phosphate binders, and iron supplements. The overall hospitalization analysis yielded a hazard ratio (HR) of 0.97 (95% confidence interval [CI]: 0.80, 1.18). A trend toward a mild protective association was observed for cardiovascular-related hospitalizations (HR: 0.85; 95% CI: 0.64, 1.14). In the subgroup analysis, a protective association was seen due to cinacalcet use for infection-related hospitalizations in the lowest intact parathyroid hormone group (HR: 0.36; 95% CI: 0.14, 0.95).

**Conclusions:**

Cinacalcet initiation in patients on maintenance hemodialysis had no effect on all-cause and cause-specific hospitalizations. Although the overall association was statistically not significant, cinacalcet may have a protective association on cardiovascular-related hospitalization in all patients and infection-related hospitalization in patient with low intact parathyroid hormone.

## Introduction

Secondary hyperparathyroidism (SHPT) is one of the most common complications that develop with chronic kidney disease (CKD) progression [1]. The prevalence of SHPT (serum intact parathyroid hormone [iPTH] > 180 pg/mL according to the Japanese guideline [2]) in dialysis patients was 32%, and the number of prevalent dialysis patients was 329,609 at the end of 2016 [3]. The risk of cardiovascular disease is markedly elevated in patients with CKD, especially those being treated with hemodialysis [4]. SHPT patients with inadequately controlled mineral metabolism abnormalities often develop high rotational bone lesions and cardiovascular calcification. Furthermore, serum phosphate (P), calcium (Ca), and intact parathyroid hormone (iPTH) concentrations have been shown to be associated with all-cause and cardiovascular mortality in dialysis patients [5,6]. In addition, dialysis patients experience high complication rates of cardiovascular diseases, infectious diseases, and vascular access (VA)-related complications. The annual risk of hospitalization lasting more than one month was 5.9-fold higher in Japanese dialysis patients compared with the Japanese general population [7].

Cinacalcet, an oral calcimimetic agent, modulates the Ca sensing receptor allosterically and increases the sensitivity of parathyroid cells to extracellular Ca ions, thereby decreasing the production and secretion of PTH and decreasing Ca and P concentrations [8]. Cinacalcet is effective for preventing the progression of vascular calcification and cardiovascular events [9–12]. Our previous analysis on the Mineral and Bone Disorder Outcomes Study for Japanese Chronic Kidney Disease Stage 5D Patients (MBD-5D) show that cinacalcet therapy improved outcomes on a composite of cardiovascular-related hospitalizations and mortality in SHPT patients with iPTH ≥ 300 pg/mL (incidence rate ratio [IRR]: 0.71; 95% confidence interval [CI]: 0.53, 0.94) [13]. These results indicate that cinacalcet may reduce cardiovascular- and VA-related hospitalizations by improving bone mineral management and inhibiting vascular calcification. However, only a few studies have specifically investigated the effectiveness of cinacalcet prescription on cause-specific and recurrent hospitalization.

Therefore, we conducted an analysis of the MBD-5D in order to elucidate the relationship between cinacalcet use and the incidence of hospitalizations in SHPT management.

## Materials and methods

### Data source and study population

MBD-5D was a 3-year, multicenter, prospective cohort study by case-cohort design. The eligibility criteria were as follows: (i) patients undergoing hemodialysis and (ii) patients with iPTH ≥ 180 pg/mL or receiving vitamin D receptor activator (VDRA) treatment. Details of the study design have been described previously [14]. Patients were randomly sampled from the whole cohort to yield a “subcohort”. The whole cohort was used to investigate mortality in a case-cohort design. Laboratory data, medication data and hospitalization outcomes were prospectively collected at 3- or 6-month intervals from patients in the subcohort. Only the patients in the subcohort were included in this analysis. Patients with cancer or cancer-related comorbidities at the time of enrollment were excluded at baseline; if a patient was diagnosed with cancer during the observation period, data obtained post-diagnosis were censored. MBD-5D was approved by the Central Ethics Committee at the Kobe University School of Medicine and was conducted in accordance with the principles of the Declaration of Helsinki. Informed consent was not mandatory according to the ethical guidelines for epidemiological research in Japan. The study is registered at ClinicalTrials.gov (NCT00995163). The reporting of this study conforms to the STROBE statement (Checklist in S1 Appendix).

This study was conducted as an ad hoc analysis of the MBD-5D study. In the original study, the sample size for the whole cohort was determined as follows: for death due to cardiovascular disease, (1) the expected rate was 2.5 deaths per 100 person-years during the 3-year follow up period, (2) the effect size of a drug was 20% to 25% of the relative risk, (3) the proportion of patients to whom the drug was prescribed was one-third. With those values, 6000–7500 patients would be required for 80% power with a two-sided alpha of 0.05 [14]. On the other hand, to examine whether a drug was associated with reduced cardiovascular hospitalization or death due to any cause in the subcohort, the sampling fraction used was 0.4 based on the following assumptions: (1) the expected rate of cardiovascular hospitalization or death due to any cause was assumed to be 13.0 events per 100 person-years during the 3-year follow-up period, (2) the effect size of a drug was assumed to be 20% to 25% of the relative risk, and (3) the proportion of patients to whom the drug was prescribed was one-third. With those values, 3000 patients would be required for 80% power with a two-sided alpha of 0.05.

### Outcomes

The primary outcome of interest was total number of all-cause hospitalizations. We additionally examined cause-specific hospitalizations for cardiovascular-, infection-, and VA-related complications, which comprise the major reasons for hospitalization in hemodialysis patients with SHPT [13]. All-cause hospitalizations were categorized into cardiovascular complications, infections, bleeding, malignant tumor, cachexia/uremia, chronic hepatitis/hepatic cirrhosis, bowel obstruction, VA complications, fractures, and other or unknown reasons. Cardiovascular-related hospitalizations were classified according to the definition guidance (S2 Appendix) and non–cardiovascular-related hospitalizations were classified based on physician’s judgment.

### Exposure

Cinacalcet use was set as the exposure of interest. To confirm the maintenance treatment effect of cinacalcet under the intention-to-treat effect observed in randomized controlled trials [15], time-varying variable describing whether each patient had initiated cinacalcet by the current time point was defined, i.e., patients contributed unexposed observations until they were initiated on cinacalcet and, once initiated, these patients contributed exposed observations regardless of their current cinacalcet use status. As the study enrollment period was prior to the market approval for cinacalcet in Japan, all patients were cinacalcet-naïve at baseline, allowing for a clear definition of cinacalcet initiation (new user design) [16, 17].

### Covariates

Clinically relevant variables that could influence subsequent cinacalcet initiation and hospitalization were considered as confounders. Time-constant confounders included demographics such as age, gender, cause of CKD, smoking status, duration of hemodialysis, history of hyperparathyroidism treatment, baseline comorbidities such as diabetes mellitus and cardiovascular disease, baseline creatinine, and baseline total protein. Time-varying medications included VDRA, phosphate binders, and iron supplements. Time-varying laboratory tests included Kt/V, iPTH, Ca, P, albumin, ferritin, iron, and hemoglobin. We obtained the time-varying covariate values 3 months prior to the time-varying exposure, which in turn was ascertained 3 months prior to the time-varying outcome to ensure the correct temporal relationship (confounders → exposure → outcome).

### Statistical analyses

Baseline continuous variables were expressed as means and standard deviations (SDs) or as medians with 25th and 75th percentiles, as appropriate. Baseline categorical variables were summarized as counts and proportions. These summaries were stratified by baseline iPTH levels (< 200 pg/mL, ≥ 200 to < 300 pg/mL, ≥ 300 to < 500 pg/mL, and ≥ 500 pg/mL; 4 strata) as well as combined iPTH-cinacalcet use strata (any cinacalcet use during the entire follow-up vs no cinacalcet use; 4 × 2 = 8 strata). The cut-offs at 300 and 500 pg/mL were set according to a previous report [13]. The cut-off value at 200 pg/mL (median iPTH for patients with iPTH < 300 pg/mL) was set in order to evaluate trends in more detail. The unadjusted changes in serum Ca, P, and iPTH were plotted at cohort enrollment and at 1, 2, and 3 years. In addition, the proportions of patients with values recommended by the 2008 Japanese guidelines for SHPT management (8.4–10.0 mg/dL for serum Ca, 3.5–6.0 mg/dL for serum P, and 60–180 pg/mL for serum iPTH) were also assessed [18]. The distribution of hospitalization outcomes among patients was described as counts by using a bar plot and was supplemented with the expected counts from a Poisson distribution with the same mean.

When the exposure is time-varying, as in our case, the confounders are also time-varying, thereby requiring special adjustments. In particular, in the setting where the time-varying confounders are affected by previous exposure (i.e., confounders are also mediators of exposure effects), the confounding effect is considered intractable by conventional methods and needs adjustment by g-methods [19], such as the marginal structural model (MSM) using inverse probability of treatment weights (IPTW) [20,21]. Our analysis used the stabilized weight construction method described in Fewell et al. [22]. Conceptually, the IPTW method constructed a weighted cohort (“pseudopopulation”) in which treatment assignment was random within the baseline covariates, making time-varying confounder adjustments in the outcome model unnecessary. Censoring was also accounted for using the inverse probability of censoring weights (IPCW). The final weight for each observation was the product of the stabilized IPTW and stabilized IPCW.

Since some patients experienced multiple hospitalizations, a recurrent-event model was used. Among the various recurrent-event models available [23,24], the simplest, Andersen-Gill model [25], was selected because more complex models incorporate time-varying confounders into the outcome modeling, which are incompatible with MSM. The Andersen-Gill outcome model was fitted using the IPTW-weighted data for each of the all-cause hospitalizations, including cardiovascular-, infection-, and VA-related hospitalizations. The analysis was initially conducted without adjusting for the interaction of exposure with baseline iPTH levels. Subsequently, the interaction was assessed to determine the potential modifying effect of cinacalcet use on hospitalization by baseline iPTH levels at cohort enrollment. Owing to the 3-month fixed interval design of the analysis, hospitalization events occasionally coincided with the visit indicated as the censoring visit and had to be dropped, as is usually done in MSMs. However, we also conducted a sensitivity analysis in which “missed hospitalizations” were retained in the dataset along with the weights that were carried forward from the observations obtained immediately before.

All analyses were performed using R version 3.4 (https://www.r-project.org/]. Missing values were imputed with multiple imputation with a chained equation [26], taking individuals as a cluster to account for the sequential nature of data points for each individual.

## Results

### Study population and baseline characteristics

**Fig 1:**
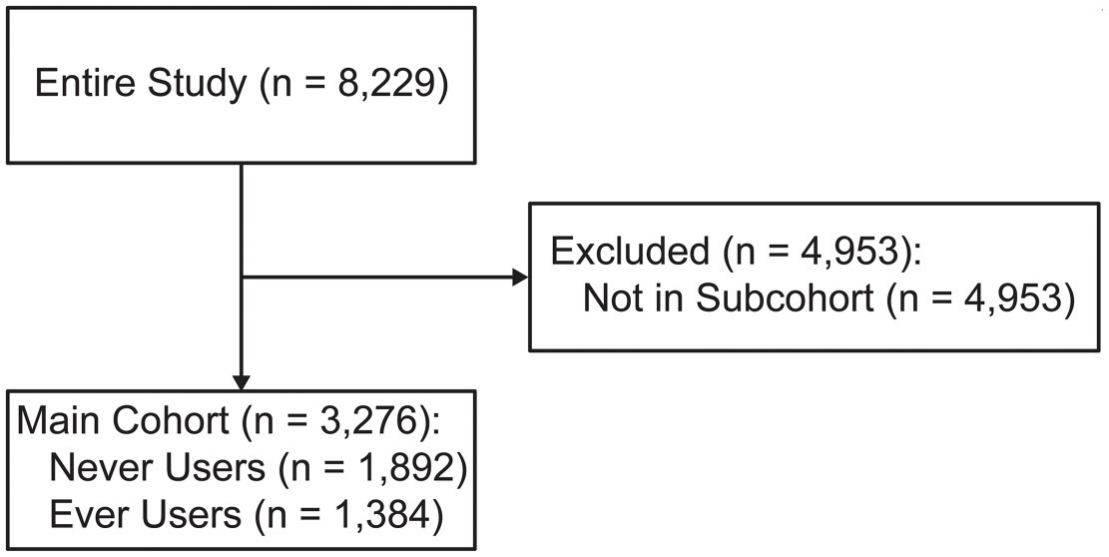
Eligibility flowchart.

A total of 3,276 hemodialysis patients with SHPT were included at cohort enrollment (Fig 1). Among these, 881 had iPTH < 200 pg/mL; 1,067 had iPTH ≥ 200 to < 300 pg/mL; 824 had iPTH ≥ 300 to < 500 pg/mL; and 504 had iPTH ≥ 500 pg/mL (Table 1). The mean age was 61.9 years, and 61.5% of the patients were male. Chronic glomerulonephritis (44.9%) and diabetic nephropathy (24.2%) were the major primary diseases. The median duration on hemodialysis was 8.3 years. S1 Table summarizes the baseline characteristics of patients who did not use cinacalcet during the entire follow-up (never users; n = 1,892) and those who use cinacalcet (ever users; n = 1,384) at some point. The ever users were younger, were on hemodialysis for a longer time, were less often diabetic, had higher serum iPTH and Ca, and were more frequently using VDRA and phosphate binders compared with the never users.

**Table 1:** Baseline characteristics of patients at study enrollment stratified by iPTH levels.

	iPTH < 200 pg/mL	200 ≤ iPTH < 300 pg/mL	300 ≤ iPTH < 500 pg/mL	iPTH ≥ 500 pg/mL	P value^a^	Overall
Number of patients	881	1,067	824	504	-	3,276
Age (mean [SD])	62.7 (12.9)	62.7 (12.6)	61.5 (12.8)	59.7 (11.9)	<0.001	61.9 (12.7)
Male (%)	62.5	62.7	59.2	60.9	0.403	61.5
Primary Disease (%)					<0.001	
Chronic Glomerulonephritis	42.1	40.8	45.1	57.9		44.9
Diabetic Nephropathy	28.9	27.9	21.1	12.9		24.2
Dialysis vintage, years (median [IQR])	6.7 (2.9, 12.4)	7.1 (3.1, 13.0)	8.9 (4.2, 15.2)	11.6 (7.4, 17.9)	<0.001	8.3 (3.7, 14.3)
BMI, kg/m^2^ (mean [SD])	21.3 (3.5)	21.4 (3.6)	21.5 (3.9)	21.2 (3.4)	0.647	21.4 (3.6)
Coronary Artery Disease (%)	26.4	26.6	24.0	23.8	0.435	25.4
Congestive Heart Failure (%)	9.3	8.2	7.3	5.8	0.123	7.9
Peripheral Vascular Disease (%)	20.3	19.1	18.4	17.7	0.625	19.1
Diabetes Mellitus (%)	36.7	35.8	29.0	18.0	<0.001	31.6
History of Parathyroidectomy (%)	4.4	5.4	6.7	10.3	<0.001	6.2
Laboratory data						
iPTH, pg/mL (median [IQR])	149.6 (97.2, 185.0)	244.0 (220.0, 271.0)	371.0 (334.0, 419.9)	691.8 (570.0, 889.2)	<0.001	265.2 (195.0, 392.0)
Calcium, mg/dL (mean [SD])	9.6 (0.9)	9.3 (0.9)	9.4 (0.9)	9.7 (0.9)	<0.001	9.5 (0.9)
Phosphate, mg/dL (mean [SD])	5.2 (1.3)	5.3 (1.3)	5.7 (1.4)	6.1 (1.5)	<0.001	5.5 (1.4)
Albumin, g/dL (mean [SD])	3.8 (0.4)	3.7 (0.4)	3.8 (0.4)	3.8 (0.4)	0.004	3.8 (0.4)
Kt/V (mean [SD])	1.4 (0.3)	1.4 (0.3)	1.4 (0.3)	1.4 (0.3)	0.090	1.4 (0.3)
Dialysate Calcium, mEq/L (mean [SD])	2.8 (0.2)	2.8 (0.2)	2.8 (0.3)	2.8 (0.3)	0.471	2.8 (0.2)
Vitamin D Receptor Agonist (%)					<0.001	
Both	0.0	0.2	0.0	0.2		0.1
Intravenous Only	58.2	35.3	45.4	65.7		48.7
Oral Only	29.6	37.1	27.1	11.9		28.7
Phosphate Binder (%)					<0.001	
Both	25.3	21.6	22.9	24.1		23.3
Non–Calcium-based Only	12.0	15.0	20.3	33.6		18.4
Calcium-based Only	49.8	46.6	41.6	29.6		43.6

BMI, body mass index; iPTH, intact parathyroid hormone; IQR, interquartile range; SD, standard deviation

^a^Differences were evaluated by one-way analysis of variance or Kruskal-Wallis test for continuous variables and by chi-square test for categorical variables.

### Descriptive statistics

The serum Ca, P, and iPTH levels stratified by baseline serum iPTH levels were plotted over time (S1 Fig). Serum Ca levels were within the recommended range (8.4–10.0 mg/dL) for 60%–70% of patients regardless of baseline iPTH levels, but not for serum P in the highest iPTH stratum (only 48% were within the recommended range [3.5–6.0 mg/dL]). By definition, the guideline values for serum iPTH were not met except in the lowest stratum. However, 30%–45% of patients regardless of baseline iPTH stratum were within the recommended range (60–180 pg/mL) at year 3.

### Outcome analysis

The IPTW numerator and denominator models both had average C-statistics of around 0.65 for predicting cinacalcet initiation. The censoring numerator model had average C-statistics of 0.73 for predicting censoring, whereas the censoring denominator model had average C-statistics of 0.80. No complete separation issues were encountered during the modeling. The average weights over time were consistently near the desired value of 1.0 regardless of the multiple imputation iteration (S2 Fig).

**Fig 2:**
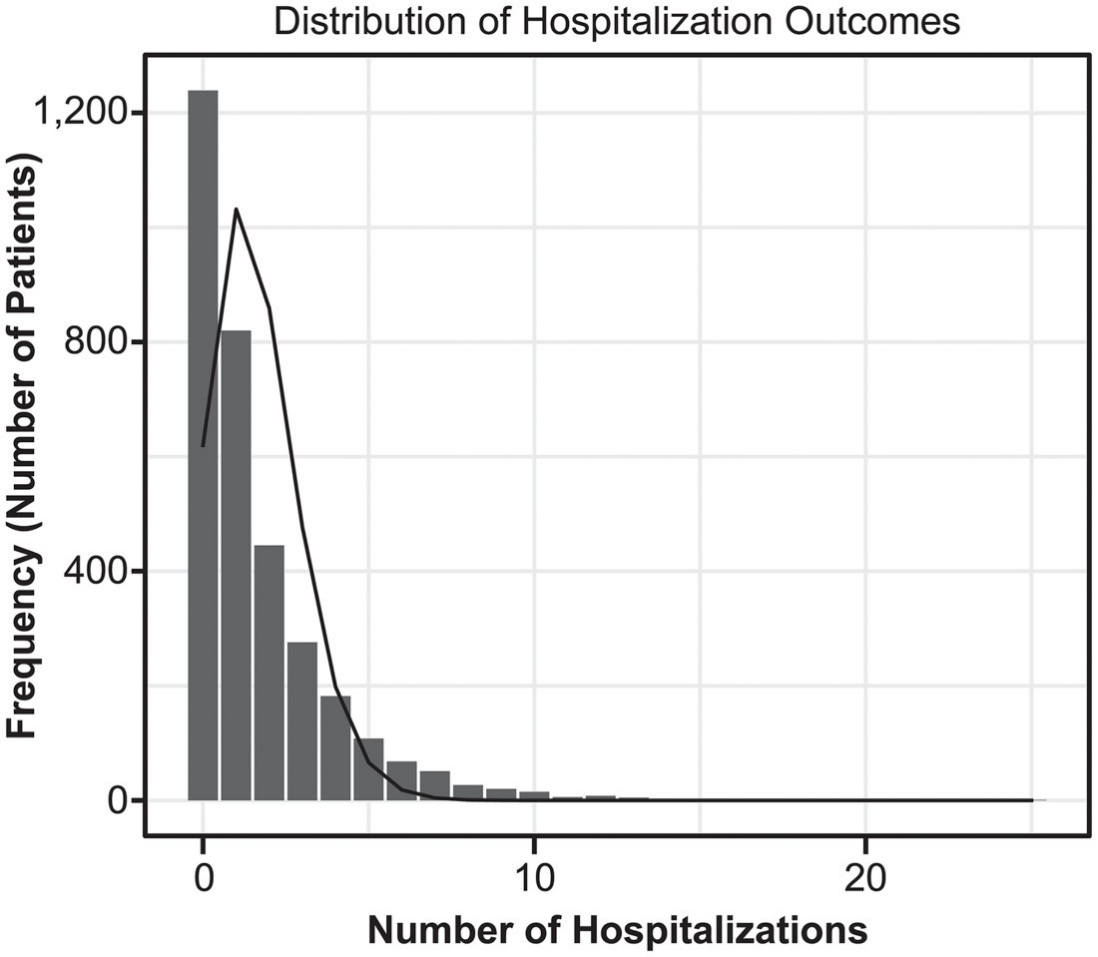
Distribution of hospitalization outcomes for each patients. The line plot indicates the counts expected from a Poisson distribution with the observed mean (1.67 hospitalizations over the study period), demonstrating the presence of overdispersion.

Of 3,276 patients in the subcohort, 656 patients were censored because of renal transplantation (n = 5), initiation of peritoneal dialysis (n = 2), death (n = 506), and loss to follow-up (n = 143). During the observation period, an average of 1.67 hospitalizations were observed (Fig 2). The major reasons for hospitalization were as follows: 22.2% cardiovascular, 14.7% VA complications, and 10.3% infectious diseases. The major causes of cardiovascular-related hospitalization were peripheral vascular disease (20.1%), angina (19.7%), cerebrovascular disease (18.3%), and congestive heart failure (14.0%).

Modeling of the exposure-outcome relationship without adjusting for the interaction between exposure and baseline iPTH levels yielded the following results: all-cause hospitalizations (hazard ratio [HR]: 0.97; 95% CI: 0.80, 1.18); cardiovascular-related hospitalizations (HR: 0.85; 95% CI: 0.64, 1.14); infection-related hospitalizations (HR: 1.01; 95% CI: 0.63, 1.63); and VA-related hospitalizations (HR: 1.01; 95% CI: 0.70, 1.46) (Table 2).

**Table 2:** Results of the outcome analysis.

Type of Hospitalization	HR	95% CI	P-value
All-cause	0.97	0.80, 1.18	0.746
Cardiovascular-Related	0.85	0.64, 1.14	0.282
Infection-Related	1.01	0.63, 1.63	0.957
Vascular Access-Related	1.01	0.70, 1.46	0.950

CI, confidence interval; HR, hazard ratio

HRs were adjusted for age, gender, cause of CKD, smoking status, duration of hemodialysis, history of hyperparathyroidism treatment, baseline comorbidities (diabetes and cardiovascular disease), baseline creatinine, baseline total protein, time-varying medications (VDRA, phosphate binders, iron supplements) and time-varying laboratory tests (Kt/V, iPTH, Ca, P, albumin, ferritin, iron, and hemoglobin).

**Fig 3:**
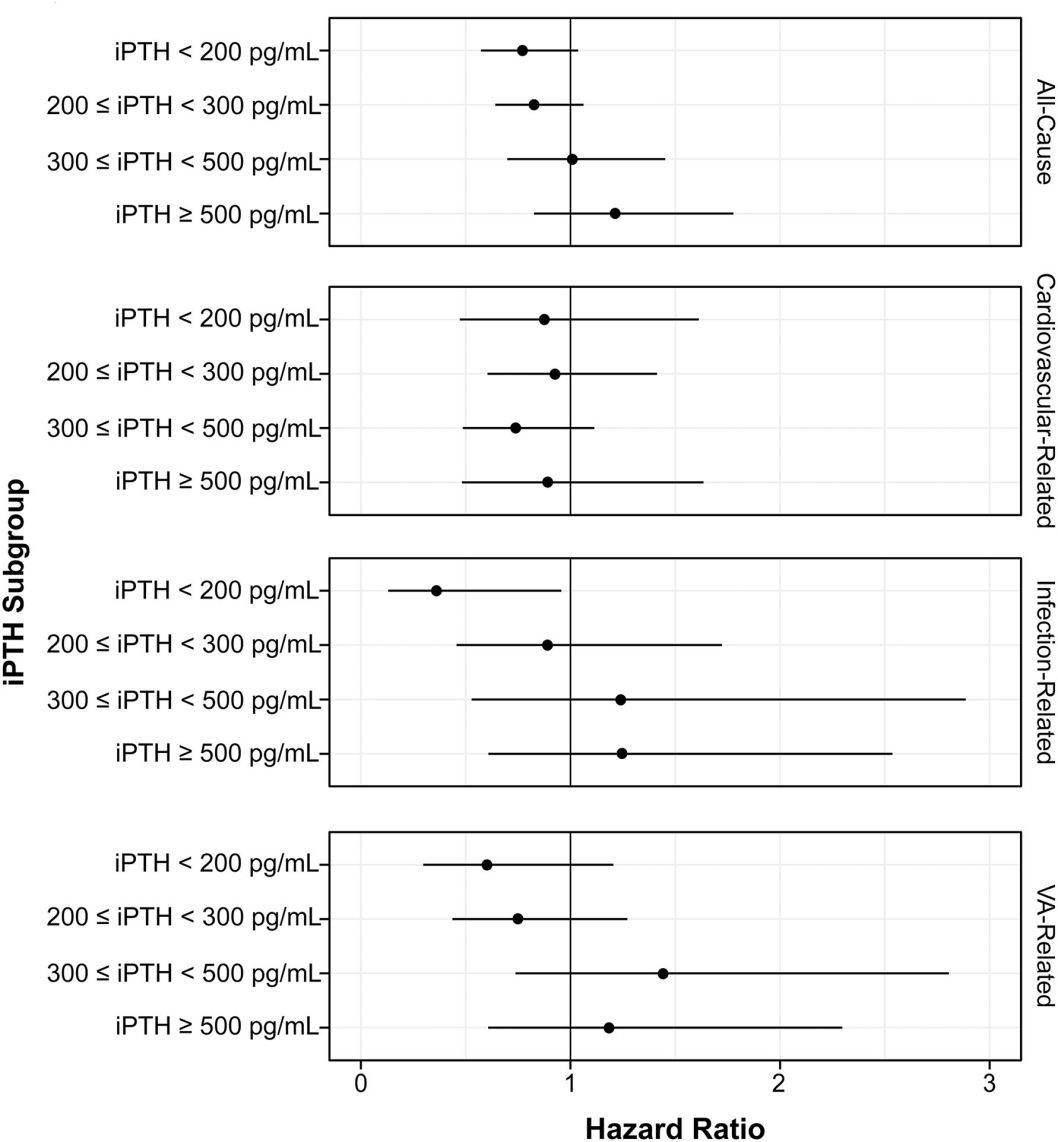
Stratum-specific effects of cinacalcet initiation on hospitalization (four-level stratification). HRs were adjusted for age, gender, cause of CKD, smoking status, duration of hemodialysis, history of hyperparathyroidism treatment, baseline comorbidities (diabetes and cardiovascular disease), baseline creatinine, baseline total protein, time-varying medications (VDRA, phosphate binders, iron supplements) and time-varying laboratory tests (Kt/V, iPTH, Ca, P, albumin, ferritin, iron, and hemoglobin). The P-values for the interaction (Wald test with three degrees of freedom) were 0.221 for all-cause hospitalizations, 0.867 for cardiovascular-related hospitalizations, 0.185 for infection-related hospitalizations, and 0.183 for VA-related hospitalizations. Abbreviations: iPTH, intact parathyroid hormone.

We then examined the interaction with baseline iPTH levels by two methods: four-level iPTH stratification (< 200, ≥ 200 to < 300, ≥ 300 to < 500, and ≥ 500 pg/mL) and two-level iPTH stratification (< 300 and ≥ 300 pg/mL). The stratum-specific associations are summarized in Fig 3 and S2 Table. A protective association was seen due to cinacalcet use for infection-related hospitalizations in the lowest iPTH group (HR: 0.36; 95% CI: 0.14, 0.95). For the cardiovascular-related hospitalizations, all point estimates were below 1.0, which suggests a consistently protective association due to cinacalcet use.

**Fig 4:**
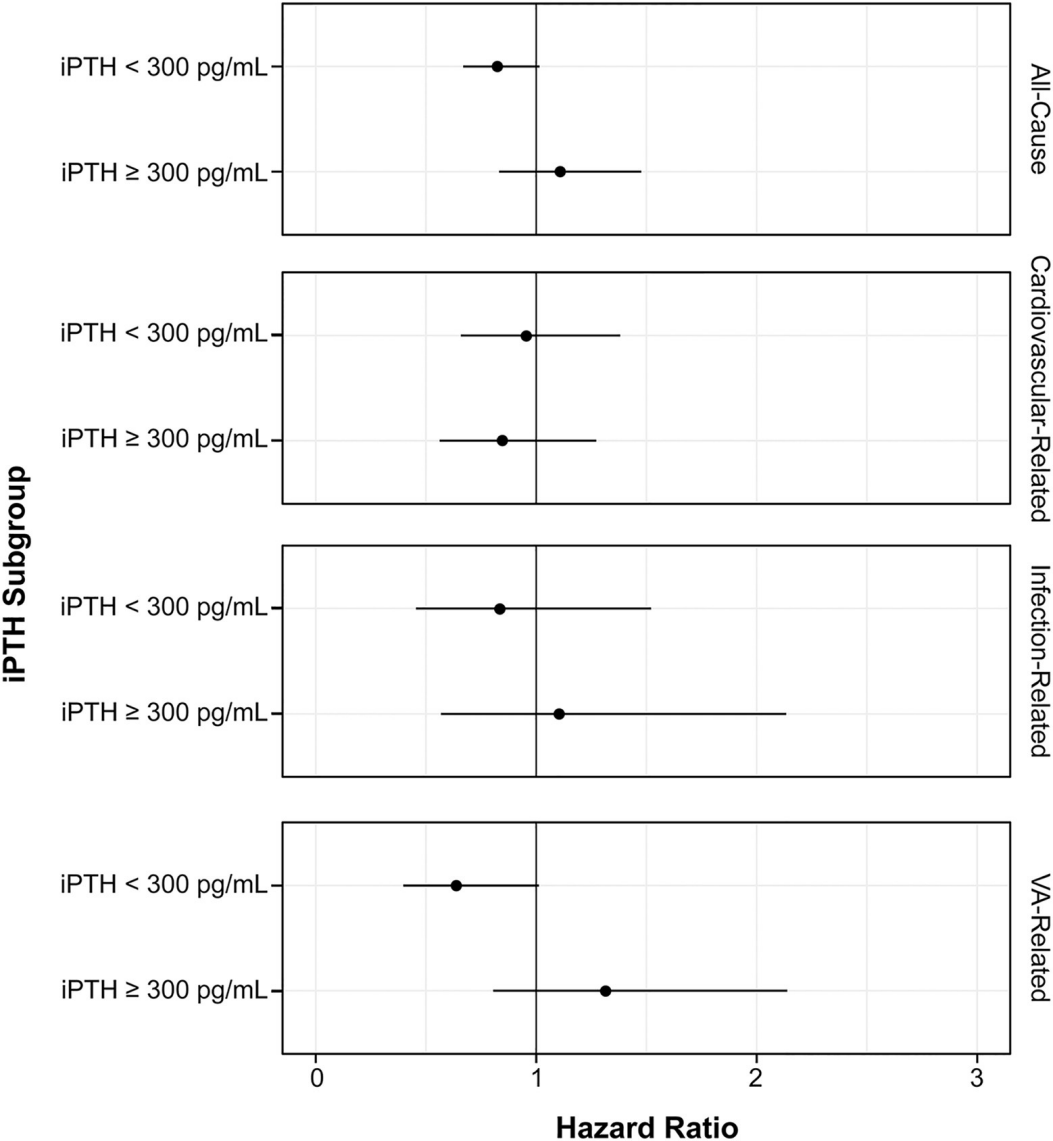
Stratum-specific effects of cinacalcet initiation on hospitalization (two-level stratification). HRs were adjusted for age, gender, cause of CKD, smoking status, duration of hemodialysis, history of hyperparathyroidism treatment, baseline comorbidities (diabetes and cardiovascular disease), baseline creatinine, baseline total protein, time-varying medications (VDRA, phosphate binders, iron supplements) and time-varying laboratory tests (Kt/V, iPTH, Ca, P, albumin, ferritin, iron, and hemoglobin). The P-values for the interaction (Wald test with one degree of freedom) were 0.077 for all-cause hospitalizations, 0.642 for cardiovascular-related hospitalizations, 0.530 for infection-related hospitalizations, and 0.024 for VA-related hospitalizations. Abbreviations: iPTH, intact parathyroid hormone.

For the two-level strata analysis, the stratum-specific associations are summarized in Fig 4 and S3 Table. The stratum-specific association of cinacalcet initiation with all-cause hospitalizations (HR: 0.82; 95% CI: 0.67, 1.01) and VA-related hospitalizations (HR: 0.64; 95% CI: 0.40, 1.01) among patients with iPTH < 300 pg/mL demonstrated a trend toward a protective association. Sensitivity analyses with the alternative censoring definitions did not change the results substantially for the overall (S4 Table) and subgroup (S5 and S6 Tables) analyses.

## Discussion

This analysis of the MBD-5D prospective cohort of hemodialysis patients aimed to elucidate the effect of cinacalcet use on all-cause and cause-specific hospitalization outcomes. During follow-up, 1,384 individuals initiated cinacalcet among the total cohort of 3,276 patients. The overall hospitalization analysis yielded an HR of 0.97 (95% CI: 0.80, 1.18). A trend toward a somewhat protective association was observed for cardiovascular-related hospitalizations (HR: 0.85; 95% CI: 0.64, 1.14), even though it is not statistically significant. In the subgroup analysis, a protective association for infection-related hospitalizations (HR: 0.36; 95% CI: 0.14, 0.95) was observed among patients with iPTH < 200 pg/mL. Although not confirmatory, these findings suggest that association of risk attenuation for cardiovascular-related hospitalizations and among patients with lower iPTH for infection-related hospitalizations were observed by cinacalcet prescription.

Several studies have reported the relationship between cinacalcet use and all-cause and cardiovascular-related hospitalizations. A pooled analysis of four randomized controlled trials with cinacalcet in dialysis patients with SHPT indicated that cinacalcet significantly reduced cardiovascular-related hospitalizations compared with the control group (HR: 0.61; 95% CI: 0.43, 0.86), but not all-cause hospitalizations [27]. The EVOLVE study indicated that there was no reduction in the composite cardiovascular endpoint with cinacalcet (HR: 0.93; 95% CI: 0.85, 1.02) in the intent-to-treat analysis [11]. The prespecified analysis adjusted for baseline parameters, parathyroidectomy, kidney transplantation, and use of commercially available cinacalcet suggests that cinacalcet significantly reduces the risk of the composite cardiovascular endpoint (HR: 0.85; 95% CI: 0.76, 0.95). Furthermore, in the recurrent events analysis of the EVOLVE study focusing on cardiovascular events, the results by the Andersen-Gill model indicated that the HRs for myocardial infarction, unstable angina, and heart failure were 0.98 (95% CI: 0.78, 1.23), 0.81 (95% CI: 0.56, 1.18), and 0.90 (95% CI: 0.72, 1.13), respectively. In this study, the HR for cardiovascular-related hospitalizations was 0.85 (95% CI: 0.64, 1.14). Although there studies included the differences in race of subject, severity of SHPT and study design, these results suggested the potential relationship between cinacalcet use and the lower incidence of cardiovascular-related hospitalizations. There are several mechanisms that cinacalcet may be associated with lower risk of cardiovascular-related hospitalizations. The first possibility is the protective effect of cinacalcet on vascular calcification [28] and cardiac hypertrophy [29]. The secondary possibility is that cinacalcet reduces circulating fibroblast growth factor 23 (FGF23) [30]. Cinacalcet treatment prevents cardiac hypertrophy via reduction in FGF23 level [31]

In this study, the impact of cinacalcet use on infection-related hospitalizations was neutral. Although there are no published reports investigating the relationship between cinacalcet use and infection-related hospitalizations, the results of a meta-analysis indicated that cinacalcet use increased upper respiratory tract infections by 79% compared with the control treatment [32]. In this study, however, no increase in infection-related hospitalizations was observed in any of the iPTH strata, and cinacalcet use was significantly associated with a lower incidence of infection-related hospitalizations in patients with the lowest iPTH. Because cinacalcet could lead to an increase in VDRA use through its serum Ca-lowering effect, it might have been associated with the lower risk of infection-related hospitalizations due to appropriate use of VDRA. It is clinically meaningful that cinacalcet did not increase infection-related hospitalizations, as avoiding infectious diseases in dialysis patients is an important concern. Similarly, no relationship between cinacalcet use and VA-related hospitalizations was observed in any patient or in most of the iPTH strata. However, a significant effect modification was observed for iPTH 300 pg/mL in both the four-level and two-level stratification. This suggests that cinacalcet use is associated with an opposite risk of VA-related hospitalizations in both iPHT patients.

The study has several limitations. First, there is a possibility of unmeasured confounding due to the nature of the analysis. Although a positive association between cinacalcet use and infection-related hospitalizations was seen in SHPT patients with iPTH < 200 pg/mL, unmeasured confounding could have been involved in this subset because of the unique patient background and the low rate of cinacalcet use. Moreover, in this study, no data were obtained on serum vitamin D, and glucocorticoid prescription. Therefore, the interaction between cinacalcet use and VA-related hospitalizations at iPTH 300 pg/mL may have been influenced by unmeasured confounding factors such as hemodynamics or vascular calcification status. Second is the possibility of masking effect due to the censoring of death in the study as competitive event. In the MBD-5D study, the adjusted IRRs of cinacalcet use for all-cause mortality in SHPT patients were 1.07 for those with iPTH < 300 pg/mL, 0.88 for iPTH ≥ 300 to < 500 pg/mL, and 0.49 for iPTH ≥ 500 pg/mL [12]. Block et al. reported that cinacalcet use showed no survival benefit in SHPT patients with iPTH < 150 pg/mL, but showed a survival benefit in SHPT patients with iPTH ≥ 600 pg/mL (HR: 0.66 [95%CI: 0.53–0.83]) [11]. We handled deaths as censored in this study and used IPCW to reduce selection bias [19]. However, it is possible that the censoring of deaths might still have biased the effectiveness of cinacalcet use on recurrent hospitalizations, especially in patients with higher iPTH, who showed an increase in HR for the all-cause, infection-related, and VA-related hospitalizations. Third, the MBD-5D study was not designed to evaluate the effectiveness of cinacalcet on case-specific hospitalizations defined in this study; therefore, it might have been underpowered. However, the sample size in this study was not less than that of past studies.

This study has several strengths. First, we used the dataset form the well-designed prospective cohort study including more than 3,000 dialysis patients with SHPT. There were no missing data for bone-mineral markers, hospitalization outcomes and covariates. Furthermore, as the study enrollment period was prior to the market approval for cinacalcet in Japan, all patients were cinacalcet-naïve at baseline. We could analyze based on a clear definition of cinacalcet initiation as new user design and adjust the potential confounders appropriately. Second, this study include the methods used for the analyses. Appropriate methods were used for handling patients with multiple occurring events and for analyzing recurrent outcomes. The average number of hospitalizations in this study was 1.67 hospitalization per patient, with the distribution indicating overdispersion. It is unlikely that each hospitalization occurred independently, suggesting that the previous hospitalization influenced the next hospitalization. The Andersen-Gill model can adjust for an event that contributes to the likelihood of occurrence of the next event, and was considered as the appropriate approach for handling multiple events in this study.

## Conclusions

Cinacalcet initiation in dialysis patients with SHPT had no effect on all-cause and cause-specific hospitalizations. Although the overall association was weak, cinacalcet may have a protective association with cardiovascular-related hospitalization in all patients and infection-related hospitalization in patient with low iPTH.

## Supporting information

S1 Table Baseline characteristics of patients at study enrollment stratified by cinacalcet use status and iPTH levels. (PDF)

S2 Table Stratum-specific effects of cinacalcet initiation on hospitalization (four-level stratification). (PDF)

S3 Table Stratum-specific effects of cinacalcet initiation on hospitalization (two-level stratification). (PDF)

S4 Table Results of the outcome analysis with the alternative censoring definition. (PDF)

S5 Table Stratum-specific effects of cinacalcet initiation on hospitalization (four-level stratification) with the alternative censoring definition. (PDF)

S6 Table Stratum-specific effects of cinacalcet initiation on hospitalization (two-level stratification) with the alternative censoring definition. (PDF)

S1 Fig Longitudinal changes in (a) serum Ca, (b) serum P, and (c) iPTH. (PDF)

S2 Fig Individual trajectories of stabilized weights. (PDF)

S1 Appendix STROBE statement. (PDF)

S2 Appendix Definitions of cardiovascular events. (PDF)

## References

[1] Komaba H, Kakuta T, Fukagawa M. Diseases of the parathyroid gland in chronic kidney disease. Clin Exp Nephrol. 2011; 15: 797–809. 10.1007/s10157-011-0502-5 21818548

[2] Fukagawa M, Yokoyama K, Koiwa F, Taniguchi M, Shoji T, Kazama JJ, et al Clinical practice guideline for the management of chronic kidney disease-mineral and bone disorder. Ther Apher Dial. 2013; 17: 247–288. 10.1111/1744-9987.12058 23735142

[3] Masakane I, Taniguchi M, Nakai S, Tsuchida K, Wada A, Ogata S, et al Annual Dialysis Data Report 2016, JSDT Renal Data Registry. Ren Replace Ther. 2018; 4: 45.

[4] Go AS, Chertow GM, Fan D, McCulloch CE, Hsu CY. Chronic kidney disease and the risks of death, cardiovascular events, and hospitalization. N Engl J Med. 2004; 351: 1296–305. 10.1056/NEJMoa041031 15385656

[5] Committee of Renal Data Registry of the Japanese Society for Dialysis Therapy. Serum phosphate and calcium should be primarily and consistently controlled in prevalent hemodialysis patients. Ther Apher Dial. 2013; 17: 221–228. 10.1111/1744-9987.12030 23551679

[6] Fukagawa M, Kido R, Komaba H, Onishi Y, Yamaguchi T, Hasegawa T, et al Abnormal mineral metabolism and mortality in hemodialysis patients with secondary hyperparathyroidism: Evidence from marginal structural models used to adjust for time-dependent confounding. Am J Kidney Dis. 2014; 63: 979–987. 10.1053/j.ajkd.2013.08.011 24119541

[7] Nishikawa K, Takahashi K, Yamada R, Kinaga T, Masato M, Yamamoto M. Influence of chronic kidney disease on hospitalization, chronic dialysis, and mortality in Japanese men: a longitudinal analysis. Clin Exp Nephrol. 2017; 21: 316–323. 10.1007/s10157-016-1293-5 27339450

[8] Nemeth EF, Steffey ME, Hammerland LG, Hung BC, Van Wagenen BC, DelMar EG, et al Calcimimetics with potent and selective activity on the parathyroid calcium receptor. Proc Natl Acad Sci USA. 1998; 95: 4040–4045. 10.1073/pnas.95.7.4040 9520489 PMC19959

[9] Komaba H, Fukagawa M. Cinacalcet and Clinical Outcomes in Dialysis. Semin Dial. 2015; 28: 594–603. 10.1111/sdi.12413 26265359

[10] Raggi P, Chertow GM, Torres PU, Csiky B, Naso A, Nossuli K, et al The ADVANCE study: a randomized study to evaluate the effects of cinacalcet plus low-dose vitamin D on vascular calcification in patients on hemodialysis. Nephrol Dial Transplant. 2011; 26: 1327–1339. 10.1093/ndt/gfq725 21148030

[11] Chertow GM, Block GA, Correa-Rotter R, Drüeke TB, Floege J, Goodman WG, et al; EVOLVE Trial Investigators: Effect of cinacalcet on cardiovascular disease in patients undergoing dialysis. N Engl J Med. 2012; 367: 2482–2494. 10.1056/NEJMoa1205624 23121374

[12] Block GA, Zaun D, Smits G, Persky M, Brillhart S, Nieman K et al Cinacalcet hydrochloride treatment significantly improves all-cause and cardiovascular survival in a large cohort of hemodialysis patients. Kidney Int. 2010; 78: 578–589. 10.1038/ki.2010.167 20555319

[13] Akizawa T, Kurita N, Mizobuchi M, Fukagawa M, Onishi Y, Yamaguchi T et al PTH-dependence of the effectiveness of cinacalcet in hemodialysis patients with secondary hyperparathyroidism. Sci Rep. 2016; 6: 19612 10.1038/srep19612 27071541 PMC4829837

[14] Fukuhara S, Akizawa T, Fukagawa M, Onishi Y, Yamaguchi T, Hasegawa T, et al Mineral and bone disorders outcomes study for Japanese chronic kidney disease stage 5D patients: rationale and study design. Ther Apher Dial. 2011; 15: 169–175. 10.1111/j.1744-9987.2010.00906.x 21426510

[15] Cole SR, Hernán MA, Margolick JB, Cohen MH, Robins JM. Marginal structural models for estimating the effect of highly active antiretroviral therapy initiation on CD4 cell count. Am J Epidemiol. 2005; 162: 471–478. 10.1093/aje/kwi216 16076835

[16] Ray WA. Evaluating medication effects outside of clinical trials: new-user designs. Am J Epidemiol. 2003; 158: 915–920. 10.1093/aje/kwg231 14585769

[17] Yoshida K, Solomon DH, Kim SC. Active-comparator design and new-user design in observational studies. Nat Rev Rheumatol. 2015; 11: 437–441. 10.1038/nrrheum.2015.30 25800216 PMC4486631

[18] Guideline Working Group, Japanese Society for Dialysis Therapy. Clinical practice guideline for the management of secondary hyperparathyroidism in chronic dialysis patients. Ther Apher Dial, 2008; 12: 514–525. 10.1111/j.1744-9987.2008.00648.x 19140852

[19] Hernan MA, Robins JM. Causal Inference [Internet]. Chapman & Hall/CRC, 2018 [cited 2 Oct 2018]. http://www.hsph.harvard.edu/miguel-hernan/causal-inference-book/

[20] Robins JM, Hernán MA, Brumback B. Marginal structural models and causal inference in epidemiology. Epidemiology. 2000; 11: 550–560. 10955408 10.1097/00001648-200009000-00011

[21] Hernán MA, Brumback B, Robins JM. Marginal structural models to estimate the causal effect of zidovudine on the survival of HIV-positive men. Epidemiology. 2000; 11: 561–570. 10955409 10.1097/00001648-200009000-00012

[22] Fewell Z, Wolfe F, Choi H, Hernán MA, Tilling K, Sterne JAC. Controlling for time-dependent confounding using marginal structural models. Stata Journal. 2004; 4: 402–420.

[23] Amorim LD, Cai J. Modelling recurrent events: a tutorial for analysis in epidemiology. Int J Epidemiol. 2015; 44: 324–333. 10.1093/ije/dyu222 25501468 PMC4339761

[24] Mieno MN, Yamaguchi T, Ohashi Y. Alternative statistical methods for estimating efficacy of interferon beta-1b for multiple sclerosis clinical trials. BMC Med Res Methodol. 2011; 11: 80 10.1186/1471-2288-11-80 21612661 PMC3118202

[25] Andersen PK, Gill RD. Cox’s Regression Model for Counting Processes: A Large Sample Study. Ann Stat. 1982; 10: 1100–1120.

[26] van Buuren S, Groothuis-Oudshoorn K. mice: Multivariate Imputation by Chained Equations in R. J Stat Softw. 2011; 45: 1–67.

[27] Cunningham J, Danese M, Olson K, Klassen P, Chertow GM. Effects of the calcimimetic cinacalcet HCl on cardiovascular disease, fracture, and health-related quality of life in secondary hyperparathyroidism. Kidney Int. 2005; 68: 1793–1800. 10.1111/j.1523-1755.2005.00596.x 16164656

[28] Raggi P, Chertow GM, Torres PU, et al The ADVANCE study: a randomized study to evaluate the effects of cinacalcet plus low-dose vitamin D on vascular calcification in patients on hemodialysis. Nephrol Dial Transplant. 2011; 26: 1327–1339. 10.1093/ndt/gfq725 21148030

[29] Choi SR, Lim JH, Kim MY, Hong YA, Chung BH, Chung S, et al Cinacalcet improves endothelial dysfunction and cardiac hypertrophy in patients on hemodialysis with secondary hyperparathyroidism. Nephron Clin Pract. 2012; 122: 1–8. 10.1159/000347145 23466553

[30] Moe SM, Chertow GM, Parfrey PS, Kubo Y, Block GA, Correa-Rotter R, et al Cinacalcet, Fibroblast Growth Factor-23, and Cardiovascular Disease in Hemodialysis: The Evaluation of Cinacalcet HCl Therapy to Lower Cardiovascular Events (EVOLVE) Trial. Circulation. 2015; 132: 27–39. 10.1161/CIRCULATIONAHA.114.013876 26059012

[31] Komaba H, Fukagawa M. The role of FGF23 in CKD- with or without klotho. Nat Rev Nephrol. 2012; 8: 484–490. 10.1038/nrneph.2012.116 22714041

[32] Zhang Q, Li M, You L, Li H, Ni L, Gu Y, et al Effects and safety of calcimimetics in end stage renal disease patients with secondary hyperparathyroidism: a meta-analysis. PLoS One. 2012; 7: e48070 10.1371/journal.pone.0048070 23133549 PMC3485048

