# Supplementary material for: Effectiveness of cinacalcet treatment for secondary hyperparathyroidism on hospitalization: Results from the MBD-5D study
Source: PLoS One. 2019 May 29;14(5):e0216399. doi: 10.1371/journal.pone.0216399 (PMC6541241; doi:10.1371/journal.pone.0216399)
Supplement: S1 Table — (PDF) [file pone.0216399.s001.pdf]

S1 Table. Baseline characteristics of patients at study enrollment stratified by cinacalcet use status and iPTH levels.

	iPTH < 200 pg/mL		200 ≤ iPTH < 300 pg/mL		300 ≤ iPTH < 500 pg/mL		iPTH ≥ 500 pg/mL		Overall		
	Never Users	Ever Users	Never Users	Ever Users	Never Users	Ever Users	Never Users	Ever Users	Never Users	Ever Users	P value[a]
**Number of patients**	631	250	693	374	404	420	164	340	1,892	1,384	-
**Age (mean [SD])**	64.2 (12.8)	58.7 (12.4)	64.5 (12.8)	59.4 (11.6)	64.0 (13.3)	59.1 (11.9)	62.6 (12.4)	58.3 (11.4)	64.1 (12.9)	58.9 (11.8)	<0.001
**Male (%)**	60.9	66.8	65.7	57.2	62.6	56.0	57.9	62.4	62.7	59.8	0.098
**Primary Disease (%)**											<0.001
**Chronic Glomerulonephritis**	38.0	52.4	36.5	48.7	36.9	53.1	56.7	58.5	38.8	53.1	
**Diabetic Nephropathy**	33.6	17.2	32.5	19.5	26.5	16.0	16.5	11.2	30.2	16.0	
**Dialysis vintage, years (median [IQR])**	5.5 (2.5, 10.8)	9.2 (5.5, 15.6)	5.1 (2.4, 10.7)	10.0 (6.4, 15.1)	5.9 (2.5, 11.0)	11.4 (7.3, 17.5)	9.5 (5.5, 14.9)	12.7 (8.2, 19.1)	5.8 (2.5, 11.4)	11.0 (6.8, 16.8)	<0.001
**BMI, kg/m^2 (mean [SD])**	21.2 (3.6)	21.5 (3.1)	21.4 (3.8)	21.3 (3.1)	21.3 (4.1)	21.6 (3.6)	20.9 (3.2)	21.4 (3.4)	21.3 (3.8)	21.5 (3.3)	0.177
**Coronary Artery Disease (%)**	27.7	23.1	28.2	23.6	25.2	22.8	29.3	21.1	27.5	22.7	0.002
**Congestive Heart Failure (%)**	9.0	10.2	9.4	6.0	9.9	4.8	7.5	5.1	9.2	6.2	0.002
**Peripheral Vascular Disease (%)**	21.3	17.9	20.2	17.2	19.5	17.3	20.5	16.4	20.5	17.2	0.021
**Diabetes Mellitus (%)**	41.7	24.2	40.9	26.3	36.0	22.2	26.4	13.9	38.9	21.6	<0.001
**History of parathyroidectomy (%)**	4.1	4.9	4.6	6.9	3.1	10.2	10.8	10.0	4.7	8.3	<0.001
**Labolatory data**											
**iPTH, pg/mL (median [IQR])**	143.0 (88.0, 185.0)	157.5 (117.9, 184.0)	240.0 (217.0, 265.2)	251.0 (227.8, 276.0)	361.5 (326.0, 408.2)	382.5 (345.1, 430.0)	680.5 (565.8, 888.3)	696.1 (573.8, 889.2)	236.3 (185.0, 324.0)	327.0 (228.5, 495.5)	<0.001
**Calcium, mg/dL (mean [SD])**	9.4 (0.8)	10.1 (0.7)	9.0 (0.8)	9.9 (0.7)	9.0 (0.8)	9.8 (0.7)	9.3 (1.0)	9.8 (0.8)	9.1 (0.8)	9.9 (0.7)	<0.001
**Phosphate, mg/dL (mean [SD])**	5.2 (1.3)	5.4 (1.2)	5.2 (1.3)	5.5 (1.2)	5.6 (1.4)	5.9 (1.3)	6.0 (1.6)	6.2 (1.5)	5.4 (1.4)	5.8 (1.3)	<0.001
**Albumin, g/dL (mean [SD])**	3.7 (0.4)	3.8 (0.4)	3.7 (0.4)	3.8 (0.4)	3.7 (0.4)	3.8 (0.4)	3.7 (0.4)	3.8 (0.4)	3.7 (0.4)	3.8 (0.4)	<0.001
**Kt/V (mean [SD])**	1.4 (0.3)	1.5 (0.3)	1.4 (0.3)	1.5 (0.3)	1.4 (0.3)	1.5 (0.3)	1.5 (0.3)	1.4 (0.3)	1.4 (0.3)	1.5 (0.3)	<0.001
**Dialysate Calcium, mEq/L (mean [SD])**	2.7 (0.2)	2.8 (0.2)	2.8 (0.2)	2.8 (0.2)	2.8 (0.2)	2.8 (0.3)	2.8 (0.3)	2.8 (0.3)	2.8 (0.2)	2.8 (0.2)	0.004
**Vitamin D Receptor Agonist (%)**											<0.001
**Both**	0.0	0.0	0.3	0.0	0.0	0.0	0.0	0.3	0.1	0.1	
**Intravenous Only**	54.5	67.6	26.3	52.1	28.7	61.4	53.7	71.5	38.6	62.5	
**Oral Only**	32.5	22.4	39.7	32.4	37.1	17.4	22.0	7.1	35.2	19.8	
**Phosphate Binder (%)**											<0.001
**Both**	20.4	37.6	17.1	29.9	19.6	26.2	22.6	24.8	19.2	28.9	
**Non–Calcium-based Only**	9.8	17.6	9.7	24.9	8.2	31.9	24.4	38.1	10.7	28.9	
**Calcium-based Only**	54.7	37.6	52.4	35.8	51.0	32.6	32.9	28.0	51.2	33.3	
**Number of hospitalization (%)**											
**All-cause**	62.4	57.6	63.6	57.5	67.6	57.4	70.7	62.6	64.7	58.7	0.001
**Cardiovascular-Related**	19.5	19.2	24.0	22.2	28.7	19.5	28.0	25.0	23.8	21.5	0.131
**Infection-Related**	12.5	6.4	15.0	12.8	18.8	11.7	9.8	9.7	14.5	10.5	0.001
**Vascular Access-Related**	14.1	13.6	13.3	15.8	14.6	13.8	10.4	13.8	13.6	14.3	0.589

BMI, body mass index; iPTH, intact parathyroid hormone; IQR, interquartile range; SD, standard deviation

[a]Differences were evaluated by unpaired t-test or Mann-Whitney U-test for continuous variables and by chi-square test for categorical variables.
