# Supplementary material for: Effectiveness of cinacalcet treatment for secondary hyperparathyroidism on hospitalization: Results from the MBD-5D study
Source: PLoS One. 2019 May 29;14(5):e0216399. doi: 10.1371/journal.pone.0216399 (PMC6541241; doi:10.1371/journal.pone.0216399)
Supplement: S2 Table — (PDF) [file pone.0216399.s002.pdf]

S2 Table. Stratum-specific effects of cinacalcet initiation on hospitalization (four-level stratification).

Type of Hospitalization	iPTH Subgroup	HR	95% CI	*P*-value
**All-Cause**	iPTH < 200 pg/mL	0.77	0.58, 1.03	0.080
**All-Cause**	200 ≤ iPTH < 300 pg/mL	0.83	0.65, 1.06	0.129
**All-Cause**	300 ≤ iPTH < 500 pg/mL	1.01	0.70, 1.45	0.961
**All-Cause**	iPTH ≥ 500 pg/mL	1.21	0.83, 1.77	0.318
**Cardiovascular-Related**	iPTH < 200 pg/mL	0.88	0.48, 1.61	0.667
**Cardiovascular-Related**	200 ≤ iPTH < 300 pg/mL	0.93	0.61, 1.41	0.719
**Cardiovascular-Related**	300 ≤ iPTH < 500 pg/mL	0.74	0.49, 1.11	0.143
**Cardiovascular-Related**	iPTH ≥ 500 pg/mL	0.89	0.49, 1.63	0.707
**Infection-Related**	iPTH < 200 pg/mL	0.36	0.14, 0.95	0.039
**Infection-Related**	200 ≤ iPTH < 300 pg/mL	0.89	0.46, 1.72	0.728
**Infection-Related**	300 ≤ iPTH < 500 pg/mL	1.24	0.53, 2.88	0.618
**Infection-Related**	iPTH ≥ 500 pg/mL	1.25	0.61, 2.53	0.543
**Vascular Access-Related**	iPTH < 200 pg/mL	0.60	0.30, 1.20	0.149
**Vascular Access-Related**	200 ≤ iPTH < 300 pg/mL	0.75	0.44, 1.27	0.280
**Vascular Access-Related**	300 ≤ iPTH < 500 pg/mL	1.44	0.74, 2.80	0.279
**Vascular Access-Related**	iPTH ≥ 500 pg/mL	1.18	0.61, 2.29	0.614

CI, confidence interval; HR, hazard ratio; iPTH, intact parathyroid hormone

HRs were adjusted for age, gender, cause of CKD, smoking status, duration of hemodialysis, history of hyperparathyroidism treatment, baseline comorbidities (diabetes and cardiovascular disease), baseline creatinine, baseline total protein, time-varying medications (VDRA, phosphate binders, iron supplements) and time-varying laboratory tests (Kt/V, iPTH, Ca, P, albumin, ferritin, iron, and hemoglobin).
