# Supplementary material for: Effectiveness of cinacalcet treatment for secondary hyperparathyroidism on hospitalization: Results from the MBD-5D study
Source: PLoS One. 2019 May 29;14(5):e0216399. doi: 10.1371/journal.pone.0216399 (PMC6541241; doi:10.1371/journal.pone.0216399)
Supplement: S4 Table — (PDF) [file pone.0216399.s004.pdf]

S4 Table. Results of the outcome analysis with the alternative censoring definition.

Type of Hospitalization	HR	95% CI	*P*-value
**All-Cause**	0.97	0.80, 1.17	0.743
**Cardiovascular-Related**	0.87	0.66, 1.15	0.340
**Infection-Related**	1.03	0.66, 1.61	0.891
**Vascular Access-Related**	1.02	0.71, 1.46	0.928

CI, confidence interval; HR, hazard ratio

HRs were adjusted for age, gender, cause of CKD, smoking status, duration of hemodialysis, history of hyperparathyroidism treatment, baseline comorbidities (diabetes and cardiovascular disease), baseline creatinine, baseline total protein, time-varying medications (VDRA, phosphate binders, iron supplements) and time-varying laboratory tests (Kt/V, iPTH, Ca, P, albumin, ferritin, iron, and hemoglobin).
