# Supplementary material for: Effectiveness of cinacalcet treatment for secondary hyperparathyroidism on hospitalization: Results from the MBD-5D study
Source: PLoS One. 2019 May 29;14(5):e0216399. doi: 10.1371/journal.pone.0216399 (PMC6541241; doi:10.1371/journal.pone.0216399)
Supplement: S1 Fig — (PDF) [file pone.0216399.s007.pdf]

S1 Fig. Longitudinal changes in (a) serum Ca, (b) serum P, and (c) iPTH.

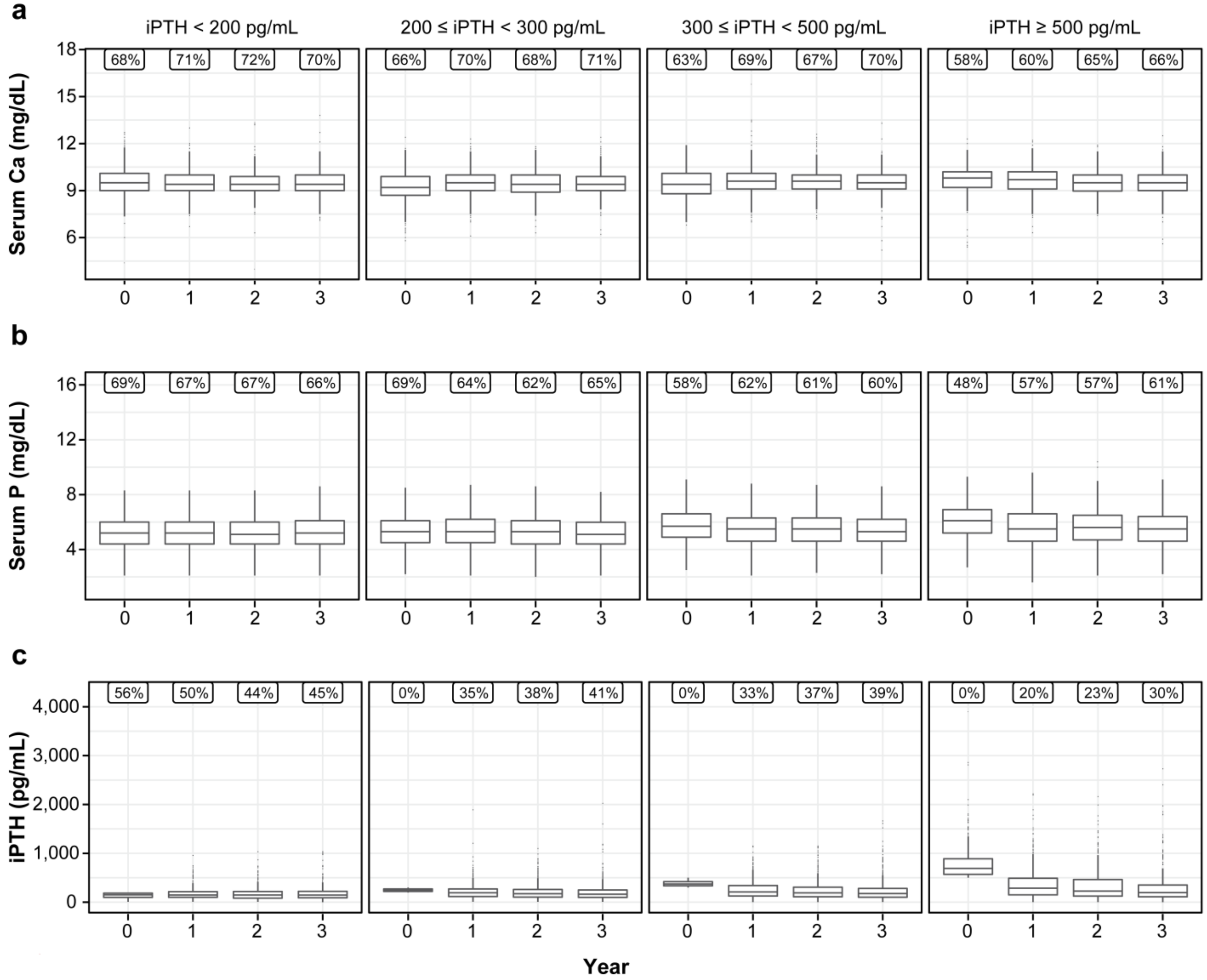

Abbreviations: Ca, calcium; iPTH, intact parathyroid hormone; P, phosphate.

Numbers shown in the rectangles are the proportion of patients meeting the 2008 Japanese guidelines for the management of secondary hyperparathyroidism. The gray horizontal areas represent the recommended target ranges, which were 8.4–10.0 mg/dL for serum Ca, 3.5–6.0 mg/dL for serum P, and 60–180 pg/mL for iPTH.
