# Supplementary material for: Effectiveness of cinacalcet treatment for secondary hyperparathyroidism on hospitalization: Results from the MBD-5D study
Source: PLoS One. 2019 May 29;14(5):e0216399. doi: 10.1371/journal.pone.0216399 (PMC6541241; doi:10.1371/journal.pone.0216399)
Supplement: S2 Fig — (PDF) [file pone.0216399.s008.pdf]

S2 Fig. Individual trajectories of stabilized weights.

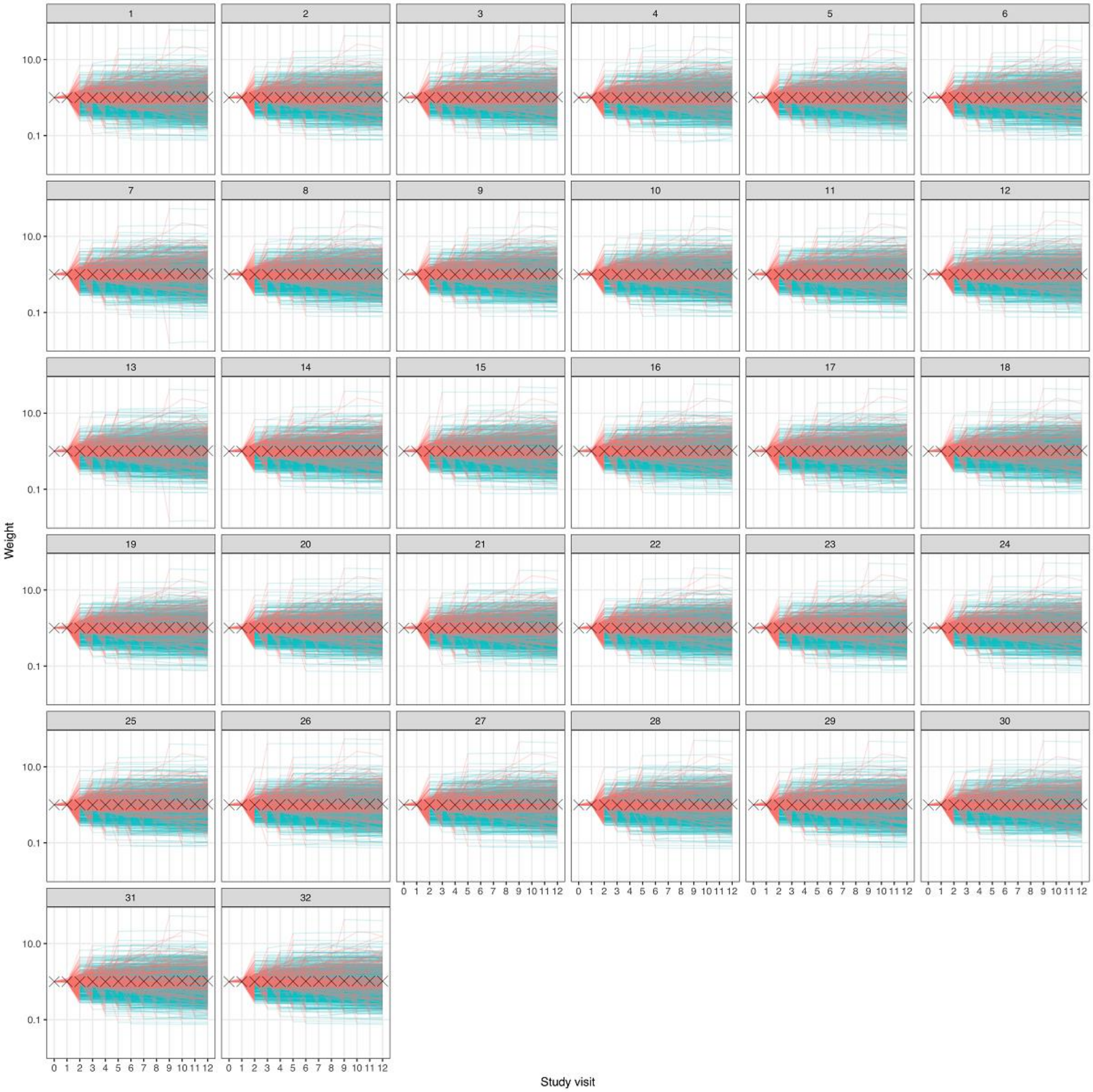

Each panel represents a single imputation dataset (32 in total). The X-axis represents the study visits. The Y-axis is the weights (log_{10} scale). All individual trajectories started in red (not initiated on cinacalcet) and changed to green once initiated on cinacalcet. The crosses (×) represent the mean at each time point, indicating that the mean was close to the ideal value of 1.0 in all datasets.
