# Supplementary material for: Effectiveness of cinacalcet treatment for secondary hyperparathyroidism on hospitalization: Results from the MBD-5D study
Source: PLoS One. 2019 May 29;14(5):e0216399. doi: 10.1371/journal.pone.0216399 (PMC6541241; doi:10.1371/journal.pone.0216399)
Supplement: S2 Appendix — (PDF) [file pone.0216399.s010.pdf]

S2 Appendix. Definitions of cardiovascular events.

1. Myocardial Infarction

Non-fatal myocardial infarction was defined as experiencing at least one of the following conditions:

(1) Electrocardiographic (ECG) abnormality fulfilling the definitive diagnostic criteria at the time of event

(2) Ischemic chest pain and elevated cardiac enzyme levels fulfilling the diagnostic criteria at the time of event

(3) Ischemic chest pain, elevated cardiac enzyme levels, and ECG abnormality fulfilling the suspected criteria at the time of event

(4) New ECG abnormality fulfilling the definitive diagnostic criteria at the time of event; however, it should not have fulfilled the definitive diagnostic criteria at the previous test

(5) Elevated cardiac enzyme

2. Angina Pectoris

(1) A case fulfilling all of the following symptoms (chest pain and chest discomfort):

- Pain, discomfort, or strangulation including any part of the sternum
- Symptoms appear at the time of exertion or stress, usually lasting over 30 seconds (the degree of exertion or stress does not matter)
- Mostly, symptoms appear within 10 minutes after relieving rest or exertion
- Symptoms usually improve within 2 to 5 minutes after nitroglycerin administration. However, this condition isn't applicable when nitroglycerin has never been administered

(2) A case without any symptoms

- Hospitalization for the diagnosis and treatment of angina pectoris within the observation period and diagnosed as angina pectoris

3. Asymptomatic Myocardial Ischemia Including Painless Myocardial Infarction

(1) New ECG abnormality suggesting myocardial ischemia

(2) A case fulfilling at least one of the following conditions:

- Myocardial ischemia has been confirmed by radioisotope examination
- Significant stenosis or occlusion of the coronary artery has been confirmed by coronary angiogram or multislice computed tomography

4. Congestive heart failure

A case fulfilling at least one of the following conditions, and clinically diagnosed as congestive heart failure:

(1) Non-acute heart failure

(2) Accompanied with marked respiratory distress

(3) Marked pulmonary edema in the chest X-ray front view

(4) Markedly high levels of brain natriuretic peptide (when measuring)

(5) Need for strict fluid balance control

5. Arrhythmia

A case with at least one of the following conditions:

(1) Ventricular fibrillation
(2) Sustained ventricular tachycardia
(3) Torsades de pointes
(4) High-grade atrioventricular block (complete atrioventricular block)
(5) Sick sinus syndrome
(6) Atrial fibrillation
(7) Atrial flutter

6. Cerebrovascular Disease

The following three types of events are targeted for diagnosing cerebrovascular disease:

(1) Cerebral infarction
(2) Cerebral hemorrhage
(3) Subarachnoid hemorrhage

For diagnosis, all of the following conditions should be fulfilled:

- Cerebral infarction, cerebral hemorrhage, or subarachnoid hemorrhage appeared recently and clear and objective focal neurological deficit observations were confirmed
- Observations lasted more than 24 hours
- Neurological observations were not due to extracranial disorders
- Cerebrovascular disease could be classified as cerebral hemorrhage, cerebral infarction, or subarachnoid hemorrhage with computed tomography or magnetic resonance imaging or autopsy records within 3 weeks of onset

7. Peripheral Artery Disease

A case fulfilling at least one of the following conditions:

(1) Vascular intermittent claudication
(2) Unilateral disappearance or weakness of the femoral artery pulse
(3) Unilateral disappearance or weakness of the dorsalis artery and posterior tibial artery pulse
(4) Disappearance or weakness the dorsalis artery and/or posterior tibial artery pulse
(5) Ankle brachial index < 0.8
(6) Angiography confirmed stenosis (more than 75% stenosis or collateral circulation)
(7) Lower leg gangrene or ulcer
